# Role of Transcription Factor EB in Mitochondrial Dysfunction of Cisplatin-Induced Acute Kidney Injury

**DOI:** 10.3390/ijms24033028

**Published:** 2023-02-03

**Authors:** Shujun Wang, Yanse Chen, Hongluan Wu, Xiaoyu Li, Haiyan Xiao, Qingjun Pan, Hua-Feng Liu

**Affiliations:** 1 Guangdong Provincial Key Laboratory of Autophagy and Major Chronic Non-Communicable Diseases, Affiliated Hospital of Guangdong Medical University, Zhanjiang 524000, China; 2 Department of Cellular Biology and Anatomy, Medical College of Georgia, Augusta University, Augusta, GA 30912, USA

**Keywords:** acute kidney injury, tubular epithelial cells, mitochondria, transcription factor EB, cisplatin

## Abstract

Cisplatin, a widely used anticancer agent, can cause nephrotoxicity, including both acute kidney injury (AKI) and chronic kidney diseases, by accumulating in renal tubular epithelial cells (TECs). Mitochondrial pathology plays an important role in the pathogenesis of AKI. Based on the regulatory role of transcription factor EB (TFEB) in mitochondria, we investigated whether TFEB is involved in cisplatin-induced TEC damage. The results show that the expression of TFEB decreased in a concentration-dependent manner in both mouse kidney tissue and HK-2 cells when treated with cisplatin. A knockdown of TFEB aggravated cisplatin-induced renal TEC injury, which was partially reversed by TFEB overexpression in HK-2 cells. It was further observed that the TFEB knockdown also exacerbated cisplatin-induced mitochondrial damage in vitro, and included the depolarization of membrane potential, mitochondrial fragmentation and swelling, and the production of reactive oxygen species. In contrast, TFEB overexpression alleviated cisplatin-induced mitochondrial damage in TECs. These findings suggest that decreased TFEB expression may be a key mechanism of mitochondrial dysfunction in cisplatin-induced AKI, and that upregulation of TFEB has the potential to act as a therapeutic target to alleviate mitochondrial dysfunction and cisplatin-induced TEC injury. This study is important for developing therapeutic strategies to manipulate mitochondria through TFEB to delay AKI progression.

## 1. Introduction

Cisplatin is the most widely used chemotherapeutic agent for various human cancers [1]. However, the use of cisplatin is limited by the side effects on normal tissues and organs, especially kidney toxicity. Approximately 33% of patients who receive cisplatin treatment develop acute kidney injury (AKI), characterized by a rapid decline in renal function [2]. Understanding the cellular and molecular mechanisms of cisplatin nephrotoxicity is important for preventing cisplatin-induced AKI. Cisplatin treatment can cause pathological renal damage, including direct renal tubular toxicity, inflammation, vascular factors, and oxidative stress [3]. Renal tubular epithelial cell (TEC) injury plays a crucial role in the initiation and progression of AKI [4,5]. Therefore, targeting the alleviation of damage to TECs could be a key strategy for preventing cisplatin-induced AKI.

Mitochondrial dysregulation has been reported as the major mechanism underlying the pathogenesis of cisplatin-induced AKI [6]. Mitochondrial dynamics were disrupted in a cisplatin-induced AKI mouse model and in cisplatin-treated HK-2 cells [7,8,9,10,11]. In addition, the level of peroxisome proliferator-activated receptor-gamma coactivator 1-alpha (PGC-1α), a transcription coactivator that regulates mitochondrial biogenesis, decreased in a cisplatin-induced AKI mouse model, suggesting that mitochondrial biogenesis was damaged [7]. Cisplatin can accumulate in the mitochondrial matrix and affect cytochrome c oxidase, leading to a decrease in or even depletion of intracellular ATP and an increase in reactive oxygen species (ROS) [12,13,14,15,16]. The increased ROS can cause mitochondrial dysfunction, thereby activating an apoptotic pathway ultimately leading to cell death [15,17,18].

Transcription factor EB (TFEB), a member of the microphthalmia family of basic helix-loop-helix–leucine-zipper (bHLH-Zip) transcription factors (MiTF/TFE family), regulates lysosomal biogenesis, function, and autophagy [19]. The protective effects of TFEB on mitochondria have recently been proven. Under normal conditions, the chemical activation of endogenous TFEB promotes the recruitment of autophagosomes to mitochondria (mitophagosome formation). When mitochondrial stress occurs, TFEB engages PINK1 and Parkin to the mitochondria, potentiating mitophagy, thereby reducing mitochondrial damage [20]. TFEB can also stabilize mitochondrial energy and function by regulating mitochondrial biogenesis and dynamics [21,22,23]. However, the effects of TFEB on mitochondria in the TECs in cisplatin-induced AKI are not yet clear. In this study, we investigated whether TFEB was involved in cisplatin-induced TEC damage and, if so, whether the protective effects were through modulation of mitochondrial function.

## 2. Results

### 2.1. Cisplatin Inhibits TFEB Expression in Mouse Kidney Tissues and HK-2 Cells

**Figure 1:**
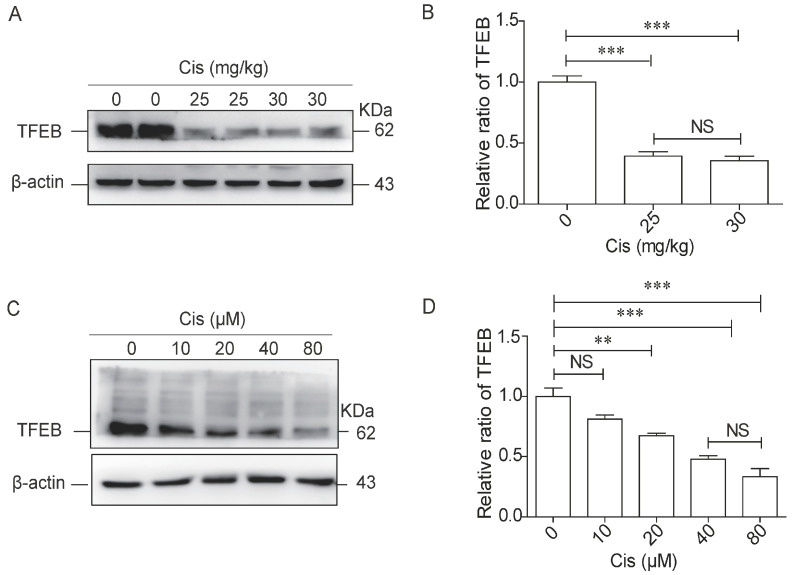
Cisplatin inhibits TFEB expression in mice kidney tissues and HK-2 cells. (**A**,**B**) Western blot assay and quantitative analysis of TFEB protein in kidney tissues of cisplatin-treated mice (intraperitoneal injection of cisplatin 0, 25, and 30 mg/kg). (**C**,**D**) Western blot assay and quantification of TFEB levels in HK-2 cells after exposure to increasing concentrations (0, 10, 20, 40, and 80 μM) of cisplatin for 24 h. Bars represent the mean ± SEM for at least three independent experiments. Cis: cisplatin. ** *p* < 0.01, *** *p* < 0.001, NS, non significant.

Based on our previous studies, cisplatin 25 mg/kg and 30 mg/kg were chosen to induce AKI in C57BL/6 mice [24]. To investigate whether cisplatin-induced kidney injury was related to TFEB, TFEB protein in TECs was examined by Western blotting. The protein levels of TFEB were significantly lower in the kidney tissue of mice treated with cisplatin at 25 and 30 mg/kg compared to the control group (Figure 1A,B). Consistent with this finding in vivo, TFEB protein levels also decreased in a dose-dependent manner in HK-2 cells after cisplatin stimulation (Figure 1C,D). These data indicate that the expression of TFEB decreased in renal tissue and cell line in a dose-dependent manner when treated with cisplatin.

### 2.2. Decreased Expression of TFEB Contributes to Renal TEC Damage Caused by Cisplatin

**Figure 2:**
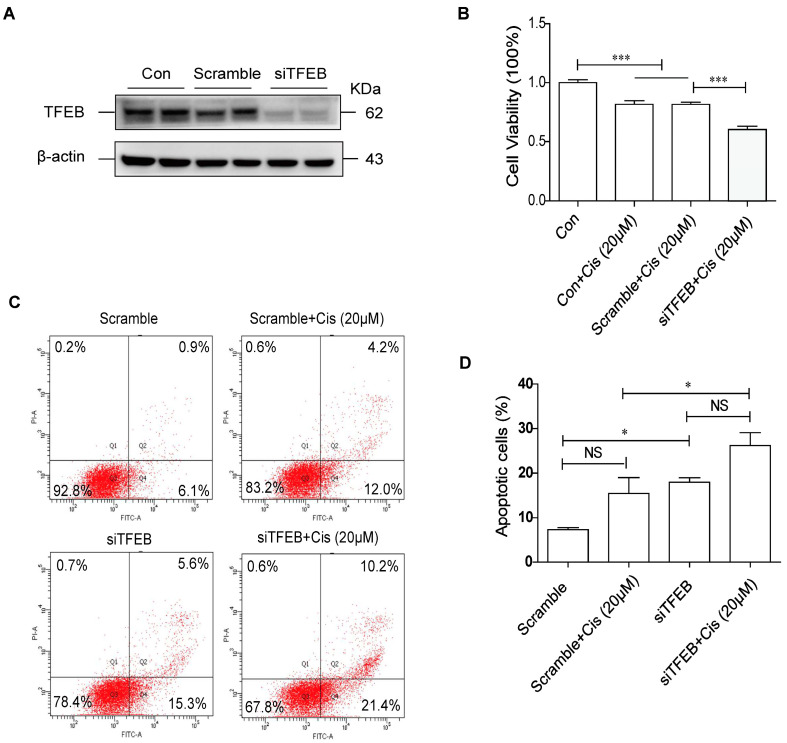
TFEB knockdown exacerbates HK-2 cells damage caused by cisplatin. (**A**) Western blot assay of TFEB expression in HK-2 cells with or without TFEB knockdown. (**B**) HK-2 cells with or without TFEB knockdown were exposed to cisplatin (20 μM) for 24 h. The CCK-8 assay was used to detect cell viability. (**C**,**D**) Flow cytometry and quantitative analysis of apoptosis in HK-2 cells. Each bar represents mean ± SEM of at least three independent experiments. siTFEB: TFEB-siRNA; Cis: cisplatin. * *p* < 0.05, *** *p* < 0.001, NS, non significant.

Since tubular apoptosis is one of the major cellular process changes in cisplatin-induced nephrotoxicity [25], we questioned whether decreased TFEB expression contributes to renal TEC damage via apoptosis. To determine the role of TFEB in cisplatin-injured TECs, HK-2 cells were transfected with TFEB-siRNA before cisplatin stimulation. TFEB protein levels significantly decreased following transfection with TFEB-siRNA (indicated as “siTFEB”) (Figure 2A). Next, the cell viability and apoptotic rate of HK-2 cells were analyzed using CCK-8 and Annexin-V/PI staining assays, respectively, after 24 h of cisplatin stimulation. The knockdown of TFEB further reduced cisplatin-induced cell viability (Figure 2B) and increased cisplatin-induced apoptosis in HK-2 cells (Figure 2C,D). These findings suggest that TFEB reduction exacerbates cisplatin-induced damage in HK-2 cells by cisplatin stimulation.

**Figure 3:**
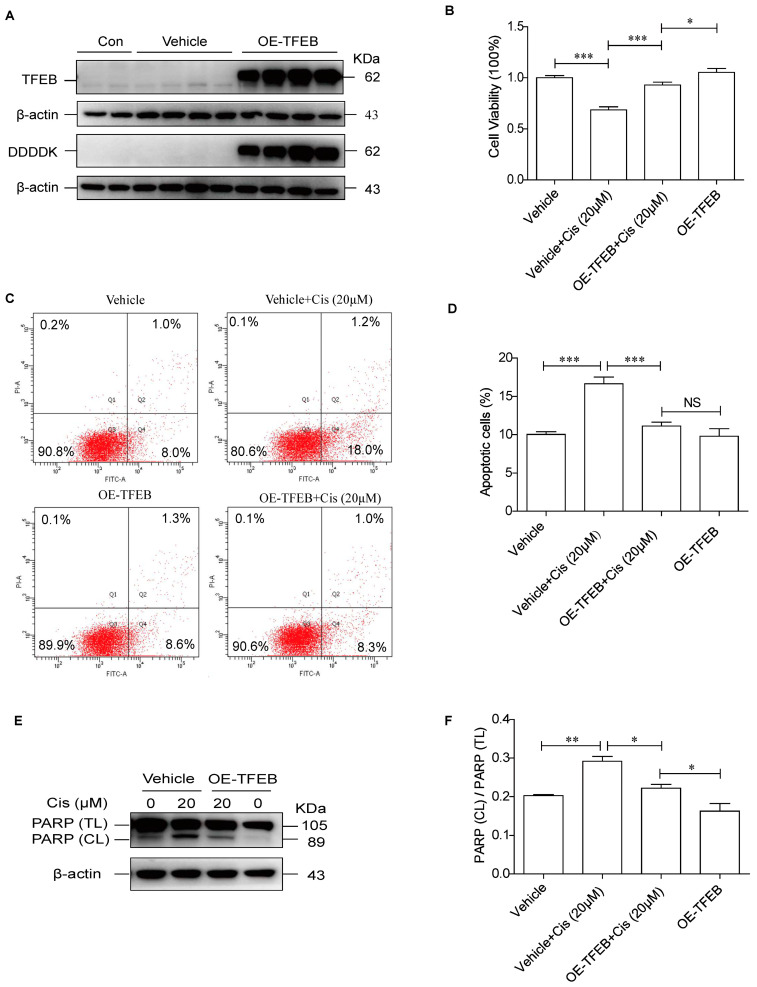
TFEB overexpression alleviates cisplatin-induced damage of HK-2 cells. (**A**) Western blot assay of TFEB expression in HK-2 cells infected with or without TFEB lentivirus. (**B**) After infection with lentivirus with or without TFEB, HK-2 cells were exposed to cisplatin (20 μM) for 24 h. The CCK-8 assay was used to detect cell viability. (**C**,**D**) Flow cytometry and quantitative analysis of apoptosis in HK-2 cells. (**E**,**F**) Immunoblotting analysis and quantitative data for PARP protein in HK-2 cells. Each bar represents mean ± SEM of at least three independent experiments. OE-TFEB: overexpressing TFEB; Cis: cisplatin. * *p* < 0.05, ** *p* < 0.01, *** *p* < 0.001, NS, non significant.

Next, we investigated whether TFEB overexpression relieved cisplatin-induced TEC damage. HK-2 cells were first infected with either a lentiviral vector expressing TFEB or an empty vector (indicated as “vehicle”). As shown in Figure 3A, TFEB overexpression effectively increased the protein levels of TFEB and DDDDK (Flag tag of TFEB). Cisplatin treatment significantly decreased cell viability, which was partially reversed by TFEB overexpression (Figure 3B). In addition, TFEB overexpression reduced cisplatin-induced apoptosis in HK-2 cells, as evidenced by reductions in the Annexin-V+/PI+ portion and decreased levels of PARP cleavage (Figure 3C–F). These findings suggest that TFEB overexpression protected TECs from cisplatin-induced injury.

### 2.3. Decreased Expression of TFEB Is Involved in Cisplatin-Induced Mitochondrial Dysfunction in TECs

**Figure 4:**
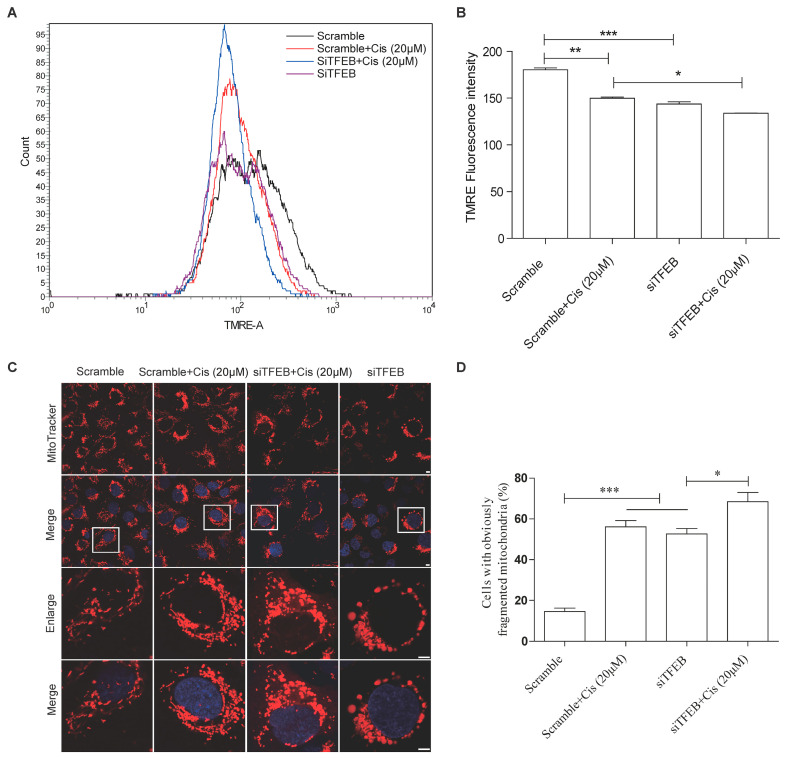
TFEB knockdown aggravates cisplatin-induced mitochondrial damage in HK-2 cells. HK-2 cells with or without TFEB knockdown were exposed to cisplatin (20 μM) for 24 h. (**A**,**B**) Flow cytometry analysis of TMRE fluorescence intensity to detect mitochondrial membrane potential in HK-2 cells. (**C**) Immunofluorescence staining with MitoTracker to detect mitochondrial morphology in HK-2 cells treated as indicated. Scale bar: 10 μm. (**D**) Percentage of cells with fragmented mitochondria in HK-2 cells. Each bar represents mean ± SEM of at least three independent experiments. siTFEB: TFEB-siRNA; Cis: cisplatin. * *p* < 0.05, ** *p* < 0.01, *** *p* < 0.001.

We further investigated the impact of TFEB on the mitochondrial system in cisplatin-treated HK-2 cells using TMRE and MitoTracker staining. After cisplatin treatment, mitochondrial membrane potential depolarization occurred, as evidenced by the loss of TMRE fluorescence intensity, which was further inhibited by TFEB knockdown (Figure 4A,B). In addition, compared with scrambled cells, the mitochondria fragmentation changed from normal strips and fusiform to short rods and spots, and the number of cells with mitochondrial fragmentation increased in cisplatin-treated HK-2 cells. TFEB knockdown significantly exacerbated cisplatin-induced mitochondrial fragmentation (Figure 4C,D), indicating that decreased TFEB expression aggravates cisplatin-induced mitochondrial lesions.

**Figure 5:**
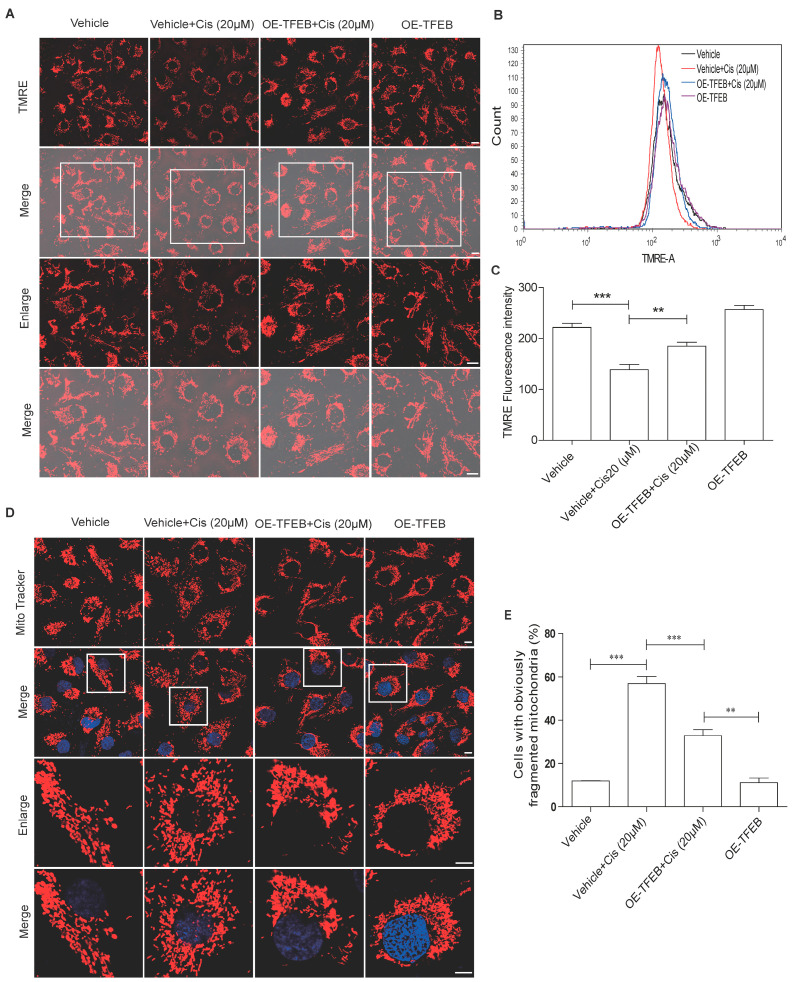
TFEB overexpression attenuates mitochondrial damage in HK-2 cells caused by cisplatin. After infection with lentivirus with or without TFEB, HK-2 cells were exposed to cisplatin (20 μM) for 24 h. (**A**) Representative images of mitochondrial membrane potential measurement with immunofluorescence staining for TMRE in HK-2 cells. Scale bar: 10 μm. (**B**,**C**) Flow cytometry analysis of TMRE fluorescence intensity to detect mitochondrial membrane potential in HK-2 cells. (**D**) Immunofluorescence staining with MitoTracker was used to detect mitochondrial morphology in HK-2 cells treated with the indicated treatments. Scale bar: 10 μm. (**E**) The percentage of cells with fragmented mitochondria in each group. Each bar represents mean ± SEM of at least three independent experiments. OE-TFEB: overexpressing TFEB; Cis: cisplatin. ** *p* < 0.01, *** *p* < 0.001.

We then tested whether TFEB overexpression could restore mitochondrial dysfunction. As shown in Figure 5A–C, HK-2 cells overexpressing TFEB partially recovered their mitochondrial membrane potential, as evidenced by the inhibition of the decrease in TMRE fluorescence intensity of HK-2 cells stimulated by cisplatin. TFEB overexpression also effectively restored the linear structure of the mitochondria and alleviated mitochondrial fragmentation in cisplatin-treated HK-2 cells (Figure 5D,E). These data suggest that TFEB overexpression could rescue cisplatin-induced mitochondrial dysfunction in HK-2 cells.

**Figure 6:**
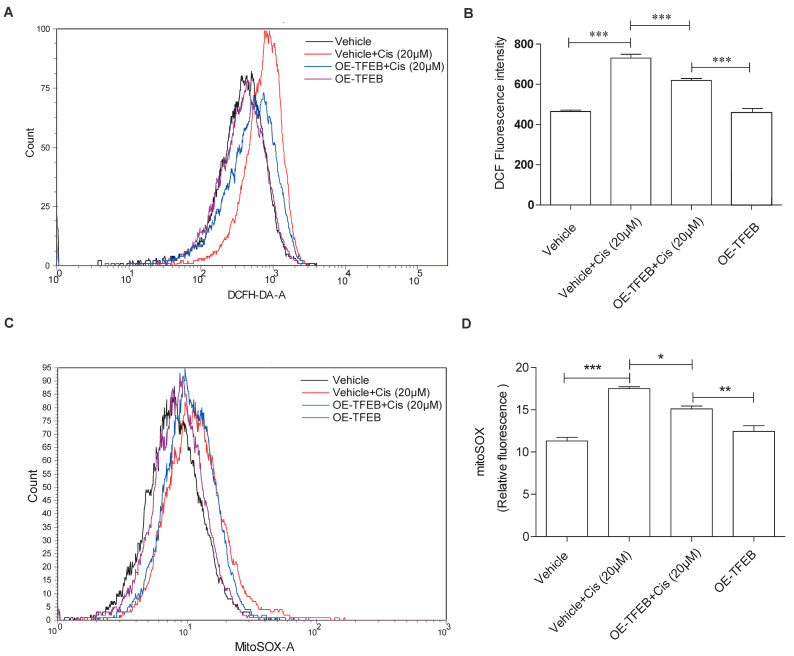
Effect of TFEB overexpression on ROS and MitoSOX in cisplatin-induced HK-2 cells. (**A**,**B**) Flow cytometry analysis and quantitative data of ROS in HK-2 cells infected by lentivirus carrying with or without TFEB followed by cisplatin (20 μM) exposure for 24 h. (**C**,**D**) Mitochondrial ROS (mtROS) were measured by incubation with MitoSOX in HK-2 cells with indicated treatments. Each bar represents the mean ± SEM for at least three independent experiments. OE-TFEB: overexpressing TFEB; Cis: cisplatin. * *p* < 0.05, ** *p* < 0.01, *** *p* < 0.001.

Overproduction of ROS contributes to mitochondrial oxidative damage [26]. We hypothesized that overexpression of TFEB may prevent oxidative damage in mitochondria. As shown in Figure 6A,B, flow cytometry results demonstrated that cisplatin-induced ROS overproduction was inhibited by TFEB overexpression. Since mitochondria are the main source of intracellular ROS [27], we also measured mitochondrial ROS (mtROS). As shown in Figure 6C,D, TFEB overexpression significantly reduced cisplatin-induced mitochondrial ROS levels in HK-2 cells. These data indicated that TFEB overexpression could rescue mitochondrial dysfunction by alleviating cisplatin-induced oxidative stress in HK-2 cells.

## 3. Discussion

Mitochondria are essential regulators of cellular energy metabolism and redox balance under stress [28]. The proximal tubules are rich in mitochondria, and maintenance of mitochondrial homeostasis is vital for kidney to function properly [29]. Mitochondrial dysfunction critically contributes to the pathogenesis of AKI [30]. However, the specific mechanisms underlying mitochondrial dysfunction have not yet been fully elucidated. In the present study, our experimental results showed that cisplatin caused mitochondrial damage in TECs. Furthermore, mitochondrial damage was mediated by the decreased TFEB expression, which was effectively rescued by TFEB overexpression.

The concentration of cisplatin in the proximal tubular cells is five times higher than in serum, suggesting that the accumulation of cisplatin in the kidney contributes to its nephrotoxicity [31]. Low concentrations of cisplatin were reported to induce apoptosis in cultured tubular cells [32]. Consistent with this finding, we observed that apoptosis was dramatically elevated in cisplatin-stimulated TECs. In addition, our data indicated that the knockdown of TFEB aggravated cisplatin-induced TEC apoptosis. Conversely, cisplatin-induced apoptosis was ameliorated when TFEB was overexpressed in those cells. These results shed light on the underlying molecular mechanisms of cisplatin-induced nephrotoxicity.

Evidence from animal experiments has demonstrated that mitochondrial damage and dysfunction are involved in cellular apoptosis in AKI [33]. In the present study, we observed depolarization of the mitochondrial membrane potential and an increase in mitochondrial fragmentation in cisplatin-stimulated HK-2 cells. A growing body of evidence suggests that TFEB is a new regulator of mitochondrial and metabolic homeostasis [34]. Mitochondrial damage caused by cisplatin was very similar to that caused by TFEB knockdown in HK-2 cells. Moreover, TFEB overexpression relieved cisplatin-induced mitochondrial damage. Therefore, the decline in TFEB expression is a key factor mediating cisplatin-induced mitochondrial damage. It is important for future research to clarify the underlying mechanism of how cisplatin stimulation decreases TFEB expression.

Excessive ROS production in HK-2 cells under cisplatin exposure can damage the mitochondria, which in turn increases the production of ROS [35]. When excessive ROS accumulate, oxidative stress occurs, triggering additional pathophysiological cascades [36]. Here, we showed that TFEB overexpression suppresses cellular ROS and mitochondrial ROS production. It has been demonstrated that the activation of TFEB removes ROS by affecting the expression of antioxidant genes and antioxidant enzyme activity [37,38,39]. These findings indicate that TFEB overexpression might protect mitochondria from damage by enhancing antioxidant ability.

In summary, these results reveal that decreased TFEB expression maybe a key mechanism of mitochondrial dysfunction in cisplatin-induced AKI, and that upregulation of TFEB has the potential to act as a therapeutic target to alleviate mitochondrial dysfunction and cisplatin-induced TEC injury. Consequently, our findings may provide evidence for developing therapeutic strategies to manipulate mitochondria through targeting TFEB to delay AKI progression.

## 4. Materials and Methods

### 4.1. Mice

Male C57BL/6 mice (7–8 weeks old; 18.0–22.0 g body weight) were purchased from the Guangdong Medical Laboratory Animal Center (Foshan, China; Quality Certificate No. 44007200050890). The mice were housed under standardized pathogen-free conditions (22 ± 2 °C) with 50 ± 10% relative humidity and a 12 h light/dark cycle at the Animal Center of Guangdong Medical University. Water and food were freely available. Mice were randomly divided into the following groups: control (CON, n = 5), cisplatin 25 mg (Cis 25, n = 5), and cisplatin 30 mg (Cis 30, n = 5). Cisplatin (Selleck Chemicals, Houston, TX, USA; S1166) was administered via intraperitoneal injection at a dose of 25 or 30 mg/kg. Mice in the control group received a single intraperitoneal injection of vehicle (saline solution). All mice were anesthetized by intraperitoneal injection of an overdose of sodium pentobarbital and euthanized at 72 h after treatment. All procedures and animal care procedures were approved by the Laboratory Animal Services Center at Guangdong Medical University (Zhanjiang, China) (SYXK-(Guangdong) 2015-0147).

### 4.2. Cell Culture and Treatments

As cultured tubular cells utilize glucose as an energy source [40], HK-2 human proximal tubular cells (ATCC, Manassas, VA, USA; CRL-2190TM) were cultured in Dulbecco’s modified Eagle’s medium (Gibco, Waltham, MA, USA; C11995500BT) supplemented with 10% fetal bovine serum (Gibco, 10270106) under standard conditions [41,42,43]. Cells were exposed to media containing different concentrations of cisplatin (0, 10, 20, 40, and 80 μM) for 24 h before being subjected to further analysis.

### 4.3. Small Interfering RNA (siRNA) Knockdown of TFEB in HK-2 Cells

siRNA oligonucleotides specific for TFEB and scrambled siRNA were obtained from GenePharma (Shanghai, China). siRNAs were transfected into HK-2 cells using the Lipofectamine™ 3000 transfection kit (Invitrogen, Carlsbad, CA, USA; L3000-015) per the manufacturer’s instructions.

### 4.4. Lentivirus and Transduction

Human TFEB (NM-007162) lentiviral particles were generated by GeneChem (Shanghai, China). The PCR-amplified full-length human TFEB gene was inserted in the AgeI/NheI sites of the GV341 pGC-FU-3FLAG-SV40-puromycin p vector (GeneChem, Shanghai, China), using the following primers: forward, 5′-CCAACTTTGTGCCAACCGGTCGCCACCATGGCGTCACGCATAGGGT-3′, and reverse, 5′-AATGCCAACTCTGAGCTTCAGCACATCGCCCTCCTCCA-3′. PSPAX2 (Addgene, Watertown, MA, USA; plasmid 12260) and PMD2.G (Addgene, plasmid 12259) were used as lentiviral packaging and envelope-expressing plasmids, respectively. An empty vector was used as a control. HK-2 cells were infected with the corresponding lentiviral supernatant for 12 h. Afterwards, the cells were cultured in complete medium for 2–3 days before being subjected to further analysis.

### 4.5. Western Blot Analysis

Total cell lysates were collected by centrifugation at 10,000× *g* for 10 min at 4 °C. After denaturation, the proteins from HK-2 cells were subjected to 12% SDS-PAGE. The resolved proteins were transferred to polyvinylidene difluoride membranes. After being blocked with 5% bovine serum albumin, the membranes were incubated with primary antibodies overnight at 4 °C with the following primary antibodies: TFEB (Bethyl Laboratories, Inc., Montgomery, TX, USA; A303-672A), poly ADP ribose polymerase (PARP, Cell Signaling Technology, Beverly, MA, USA; 9532), DDDDK (Abcam, Cambridge, UK; Ab205606), and β-actin (Santa Cruz Biotechnology, Dallas, TX, USA; Sc47778), followed by incubation with the secondary antibody for 1 h at room temperature (22–28 °C). Proteins were analyzed using Image-Pro Plus version 6.0 software (Media Cybernetics, Rockville, MD, USA).

### 4.6. Cell Viability

Cells (2 × 10^5^) were cultured in wells of a 96-well plate. Following stimulation, cell viability was examined using Cell Counting Kit-8 (CCK-8; Beyotime, Shanghai, China; C0039). According to the manufacturer’s instructions, 10 µL of CCK-8 solution was added to each well containing 100 µL of medium. The absorbance was measured at 450 nm after incubating for 1.5 h.

### 4.7. Fluorescein Isothiocyanate (FITC) Annexin-V/Propidium Iodide (PI) Assay for HK-2 Cell Apoptosis

Cells were stained using the Annexin-V/PI Apoptosis Detection Kit (Dojindo Laboratories, Kumamoto, Japan; AD10) according to the manufacturer’s protocols before being analyzed by flow cytometry using the FACSCanto II platform (BD Biosciences, Santa Clara, CA, USA).

### 4.8. Detection of Mitochondrial Membrane Potential

Following stimulation, the cells were washed twice with PBS, and 70 nM tetramethylrhodamine (TMRE, Invitrogen; T669) was added according to the manufacturer’s protocol. The cells were incubated in the dark at 37 °C for 10 min. After incubation, the cells were washed three times and examined by flow cytometry or photographed immediately using a model FV3000 confocal microscope (Olympus, Tokyo, Japan) at ×630 magnification.

### 4.9. Intracellular ROS Detection

Cells (1 × 10^5^) were cultured in wells of 12-well plates and washed three times with PBS following stimulation. The fluorescent probe 2’,7’-dichlorodihydrofluorescein diacetate (DCFH-DA, Invitrogen; C6827) was added to all wells. The cells were incubated in the dark for 10 min at 37 °C and washed with PBS. Cells were collected into a flow tube and examined using a FACS-Aria II flow cytometer (BD Biosciences) within 1 h. FCS Express V3 software was used for the analysis.

### 4.10. Mitochondrial ROS Detection

After washing twice with pre-warmed Hanks’ balanced salt solution/Ca/Mg buffer, cells were incubated in the dark with MitoSOX reagent (5 µM, Invitrogen; M36008) working solution at 37 °C for 12 min. The cells were examined by flow cytometry or photographed immediately with a confocal microscope (Leica Microsystems GmbH, Wetzlar, Germany) at ×630 magnification after being washed twice with Hanks’ buffer.

### 4.11. Mitochondrial Morphology Detection

Cells were seeded and grown on glass coverslips. After incubating the cells with 100 nM MitoTracker Red (Invitrogen; M7512) at 37 °C for 10 min, mitochondrial morphology was visualized, and images were acquired using a confocal laser scanning microscope with 63× oil immersion objective lens.

### 4.12. Statistical Analysis

The results are presented as mean ± SEM of at least three independent experiments. Data were analyzed using SPSS version 23.0 software (SPSS, Inc., Chicago, IL, USA) and Prism version 6.0 software (GraphPad, San Diego, CA, USA). One-way analysis of variance (ANOVA) was used to compare the differences between groups. Statistical significance was set at *p* < 0.05.

## 5. Conclusions

These findings suggest that tubular mitochondrial dysfunction in cisplatin-induced AKI is mediated, at least partially, by decreasing TFEB expression, and that upregulation of TFEB expression could be a potential therapeutic target for the alleviation of mitochondrial dysfunction and cisplatin-induced TEC injury.

## Data Availability

The data that support the findings of this study are available on request from the authors.
